# Sorting Nexin 6 Enhances Lamin A Synthesis and Incorporation into the Nuclear Envelope

**DOI:** 10.1371/journal.pone.0115571

**Published:** 2014-12-23

**Authors:** Jose M. González-Granado, Ana Navarro-Puche, Pedro Molina-Sanchez, Marta Blanco-Berrocal, Rosa Viana, Jaime Font de Mora, Vicente Andrés

**Affiliations:** 1 Department of Atherothrombosis, Imaging and Epidemiology, Centro Nacional de Investigaciones Cardiovasculares (CNIC), Madrid, Spain; 2 Instituto de Biomedicina de Valencia (IBV), Consejo Superior de Investigaciones Científicas, Valencia, Spain; 3 Fundación para la Investigación Hospital La Fe, and Instituto Valenciano de Patología, Facultad de Medicina, Universidad Católica de Valencia San Vicente Mártir, Valencia, Spain; University of the Basque Country, Spain

## Abstract

Nuclear lamins are important structural and functional proteins in mammalian cells, but little is known about the mechanisms and cofactors that regulate their traffic into the nucleus. Here, we demonstrate that trafficking of lamin A, but not lamin B1, and its assembly into the nuclear envelope are regulated by sorting nexin 6 (SNX6), a major component of the retromer that targets proteins and other molecules to specific subcellular locations. SNX6 interacts with lamin A *in vitro* and *in vivo* and links it to the outer surface of the endoplasmic reticulum in human and mouse cells. SNX6 transports its lamin A cargo to the nuclear envelope in a process that takes several hours. Lamin A protein levels in the nucleus augment or decrease, respectively, upon gain or loss of SNX6 function. We further show that SNX6-dependent lamin A nuclear import occurs across the nuclear pore complex via a RAN-GTP-dependent mechanism. These results identify SNX6 as a key regulator of lamin A synthesis and incorporation into the nuclear envelope.

## Introduction

Polymers of A- and B-type lamins interact with proteins anchored in the nuclear membrane to form the perinuclear lamina of mammalian cells [1]. This complex structure ensures the correct assembly of the nuclear envelope (NE) and regulates multiple cellular functions, including chromatin organization, signal transduction and gene expression [2]–[4].

The NE consists of the inner and outer nuclear membranes (INM and ONM) and the intervening perinuclear space with the nuclear pore complexes (NPCs) mediating active and passive transport of molecules between the cytoplasm and nucleus, and the nuclear lamina covering the INM, which contains A- and B-type lamins [5], [6]. The NE is fused to the endoplasmic reticulum (ER) and shares some of its properties, and indeed is considered to be a specialized ER domain [7]. Four non-exclusive models have been proposed for the transport to the INM of the proteins that maintain NE homeostasis in interphase cells: (1) diffusion-retention, (2) targeting with classical nuclear localization signal (NLS), (3) vesicle fusion, and (4) targeting with specific INM-sorting motifs [8], [9]. 1) The diffusion-retention model suggests that integral membrane proteins synthesized in the ER reach the ONM by diffusion [10] and then transfer to the INM by passive lateral diffusion at sites of NPC insertion [11]. 2) The NLS model proposes that an NLS in proteins destined for the INM is recognized by importins and karyopherins, which then interact with the NPCs, resulting in transport of INM proteins to the nuclear interior along gradients of soluble Ran-GTP/Ran-GDP created by Ran-GTPases [12], [13]. 3) The vesicle fusion model is supported by studies showing that depletion of vesicle-fusion regulators impairs NE formation [14]. 4) Targeting with specific INM-sorting motifs is an active transport mechanism in which importin-α-16, a truncated form of importin-α, recognizes INM-sorting motifs in proteins at the ER and facilitates their transport into the nucleus [15], [16].

The endosomal pathway is responsible for plasma membrane cargo uptake and sorting. Cell-surface receptor tyrosine kinases that undergo endocytosis are subsequently fused with early endosomes and then translocated to the nucleus [17]–[19]. Retrograde transport of transmembrane proteins from endosomes to the transGolgi network is mediated by the retromer, a heteropentameric complex that associates with the cytosolic surface of endosomes [20]. The retromer is composed of a vacuolar protein sorting trimer and a sorting nexin (SNX) dimer, which is responsible for binding to specific phosphoinositides [21], [22] and for the formation of high curvature membrane tubules [23], [24]. Localized extreme membrane curvature also requires content of specific lipids such as diacylglycerol [25]. ER tubules physically contact and encircle endosomes while they traffic and mature [26]. Retrograde transport is altered in a number of human infectious diseases [27], [28], as well as in Alzheimer's disease [29], cancer [30], and possibly in osteoporosis [31].

Nuclear import of soluble proteins larger than 40 kDa and shuttling of proteins to the nuclear interior against a concentration gradient requires active transport through the NPC [32]. Transit of integral membrane proteins from the ER to the INM is also energy-dependent [33] and requires interaction with other proteins [15], [34], [35]. Early sorting of INM proteins is highly conserved [16], suggesting a fundamental role in NE homeostasis; however, little is known about the precise mechanism by which A-type lamins incorporate into the nuclear lamina and how this process is influenced by other trafficking proteins. Here, we show that lamin A synthesis and nuclear import are regulated by SNX6 through a RAN-GTP-dependent mechanism.

## Materials and Methods

### Plasmids

The following plasmids were as described previously: pECFP-Lamin A [36]; pECFP-SNX6, pEYFP-SNX6 and pGEX4T3-GST-SNX6 [37]; FLAG-prelamin A [38]; HA-Lamin A [39]; mRFP1-Sec-61beta [40]; pcDNA3-eNOS-GFP [41]; Rtn3-tdTomato [42]; pEGFP-lamin A [43]. YFP-Lamin B1 and CFP-Lamin B1 were obtained from EUROSCARF and RANQ69L from Addgene (plasmid 30309) [44]. HA-SNX6 was obtained from Dr. J.S. Bonifacino (Shriver National Institute of Child Health and Human Development, NIH, USA), pEGFP-Lamin C from Dr. D. Pérez-Sala (Centro de Investigaciones Biológicas, CSIC, Spain), and pEYFP-FrataxinMLS from Dr. F. Palau (Instituto de Biomedicina de Valencia, CSIC, Spain).

### Cell culture

U20S cells were obtained from the American Type Culture Collection. Cells were incubated at 37°C in a 5% CO_2_/95%O_2_ atmosphere and maintained in DMEM supplemented with 100 U/ml penicillin, 0.1 mg/ml streptomycin, and 2 mmol/L L- glutamine (Invitrogen) and 10% FBS. Mouse primary SMCs were isolated from aortas harvested from 3-month-old wild-type mice after two digestions in HBSS/fungizone medium. Briefly, the mouse aorta was first digested with type II collagenase (175 U/ml) (ref. 4176, Worthington Biochemical Corp., Lakewood, New Jersey, USA) to remove the adventitia and SMC suspensions were obtained after a second digestion with type II collagenase (175 U/ml) and type I elastase (4.7 U/ml) (ref. 45124, Sigma). Mouse SMCs were initially cultured in DMEM with 20% FBS and 1% fungizone/penicillin/streptomycin/glutamine, and afterwards as described above. *Lmna*-null mouse embryonic fibroblasts (MEFs) are described elsewhere [45].

### Antibodies

The monoclonal anti-SNX6 antibody 446A was used for immunoblotting studies [37]. Other primary antibodies were acquired from the following providers: anti-GST (sc-138), anti-lamin A/C (sc-6215), anti-ERK2 (sc-1647), anti-tubulin (sc-8035), anti-UCP2 (sc-6526), anti-lamin A (sc-20680) anti-SP1 (sc-59-G), and anti-GRP94 (sc-11402) from Santa Cruz Biotechnologies; anti-HA (H-9658) and anti-Flag (F-3165) from Sigma; anti-early endosome antigen 1 (EEA1) (ab14453) from Abcam; anti-GFP (A6455) from Invitrogen; and anti-p27 (610242) from BD Transduction Laboratories. Isotype-specific HRP-coupled secondary antibodies were from Santa Cruz Biotechnology.

### RNA interference

Transient silencing of SNX6 with a pcDNA 6.2-GW miR plasmid encoding a mi-RNAi against SNX6 (Invitrogen) was performed as previously described [37].

### GST pulldown assays

GST and GST-SNX6 proteins were purified using glutathione-Sepharose 4B (Amersham Biosciences Corp., Piscataway, NJ, USA) and were eluted with 50 mM Tris-HCl (pH 8.0) and 10 mM glutathione. Whole cell extracts were prepared by sonication in ice-cold lysis buffer (20 mM Tris-HCl at pH 7.0, 1% NP-40, 150 mM NaCl, 10% glycerol, 10 mM EDTA, 20 mM NaF, 5 mM sodium pyrophosphate, 1 mM Na_3_VO_4_, 1 mM PMSF). Whole extracts (500 µg) of transfected U2OS cells (overexpressing HA-lamin A) or of mouse SMCs were incubated with recombinant proteins (3 µg) in RIPA buffer (150 mM NaCl, 1% Nonidet P-40, 0.5% sodium deoxycholate, 0.1% SDS, and 50 mM Tris, pH 8) supplemented with protease inhibitor cocktail (Complete; Roche Diagnostics, Indianapolis, IN, USA). After overnight incubation at 4°C, glutathione-Sepharose 4B was added to a final concentration of 10%, and the samples were agitated at 4°C for 1 h. Beads were collected by centrifugation and washed three times with RIPA buffer. Pellets were air-dried, resuspended in 2 µ Laemmli buffer, boiled for 5 min, and separated by 12% SDS-PAGE.

### FRET

U2OS cells were cotransfected with pEYFP-SNX6 and pECFP-Lamin A or with pEYFP and pECFP-Lamin A as a negative control (4 µg each plasmid, calcium phosphate method). Images were acquired on a Leica TCS/SP2 confocal microscope with a 63× oil immersion objective (NA 1.4). An argon laser line of 458 nm was used to excite CFP (PMT window 465–505 nm) and a 514-nm line to excite YFP (20% laser intensity for acquisition, and 65% for photobleaching) (PMT window 525–600 nm). FRET studies were performed with 4% PFA-fixed cells using the acceptor-photobleaching method as previously described [36], [37], [46]. Briefly, FRET was calculated as the relative increase in donor fluorescence resulting from the reduction or elimination of energy transfer when the acceptor YFP is photobleached. The percentage of pixels exhibiting increased CFP fluorescence intensity after photobleaching was quantified in the regions of interest using the following equation:

FRET efficiency  = (C_after_-C_before_)/C_after_ ×100, where C_before_ and C_after_ are the total fluorescence intensities (area × average intensity of bright points) of the CFP channel before and after photobleaching, respectively.

### Permeabilization assays

U2OS cells were transfected using the calcium phosphate method with plasmids encoding GFP-Lamin A and HA-SNX6 (4 µg each plasmid). After 24 hours, cells were fixed with 4% PFA. When indicated, cells were treated for 30 min at room temperature with digitonin (40 µg/ml of PBS, to permeabilize the plasma membrane), Triton-X100 (0.5% in PBS, to permeabilize all membranes) or PBS (no permeabilization). To visualize lamin A/C, cells were incubated with anti-GFP or anti lamin A/C antibodies for 1 h at room temperature followed by incubation with appropriate Alexa-647-labelled secondary antibodies (45 min, room temperature) as described below.

### Confocal microscopy

For all immunofluorescence experiments different from permeabilization assays, non-transfected cells (control) and transfected cells (calcium phosphate method) were grown on glass coverslips. All procedures for immunostaining were performed at room temperature. Cells were first fixed for 15 min with 4% PFA/PBS, and permeabilized with 0.5% Triton X-100 for 15 min. Cells were then treated with 10 mM glycine (pH 8.5) for 5 min, blocked for 1 h with 5% dry milk (dissolved in 10% FBS/0.5% BSA/0.1% Triton X-100/PBS), and incubated for 1 h with primary antibodies (anti-lamin A/C, anti-GFP, anti HA or anti-FLAG) followed by 45 min with appropriate Alexa488- or Alexa647-labeled secondary antibodies (Molecular Probes). To visualize mitochondria, cells were transfected with a plasmid encoding the mitochondrial localization signal (MLS) of frataxin bound to YFP (pEYFP-FrataxinMLS). To visualize the Golgi apparatus, cells were transfected with pcDNA3-eNOS-GFP or treated with Bodipy TR ceramide (Invitrogen) according to the manufacturer's instructions.

Cells were examined under a Leica TCS/SP5 laser confocal microscope fitted with an HCX PL APO 63/NA 1.40-0.60 oil immersion objective, under a Leica TCS/SP2 laser confocal microscope fitted with a 63× oil immersion objective (NA 1.4), or under a NIKON A1-R inverted confocal microscope fitted with an 60× oil immersion objetive (NA 1.4). Filters were used for detection of DAPI, CFP, GFP, YFP, and Texas red. In live imaging experiments, microscopes were covered by a full acrylic box allowing live-cell imaging at 37°C, 5% CO_2_. Images were analyzed with Leica LASAF (Leica Microsystems), Metamorph (Molecular Devices), Imaris (Bitplane) or ImageJ (NIH). Cells with aberrant (extranuclear) endogenous or exogenous lamin A or lamin A/C distributions was calculated as the percentage of cells with at least one spot of lamin A or lamin A/C outside the nucleus and corrected for the number of transfected or total cells.

### Time-lapse fluorescence confocal microscopy

U2OS cells cotransfected with GFP-Lamin A and HA-SNX6 were examined under a Nikon ECLIPSE Ti time-lapse inverted microscope fitted with an 40× air objective (NA 0.6) using filters for GFP and Cy3. U2OS cells cotransfected with RFP-Sec-61, GFP-Lamin A and HA-SNX6 were examined under a TCS SP5 confocal laser scanning unit attached to an inverted epifluorescence microscope (DMI6000) fitted with an HCX PL APO 63/NA 1.40-0.60 oil objective. Cells were maintained in DMEM (containing 10%FBS and 20 mM Hepes) in 35 mm dishes (MatTek) at 37°C in a 5% CO_2_ atmosphere.

### Quantitative real-time PCR (RT-qPCR)

Total RNA from U2OS cells transfected with YFP or YFPSNX6 was isolated with Qiazol Lysis Reagent (Qiagen, Valencia, CA) and isopropanol precipitation, or with the RNeasy Mini kit according to the manufacturer's instructions (Qiagen). RNA concentration and purity were assessed from the A260 nm/A280 nm ratio and integrity was verified by separation on ethidium bromide-stained 1% agarose gels. cDNA was generated from total RNA (0.1–1 µg) using the High Capacity cDNA Reverse Transcription Kit (Applied Biosystems, Foster City, CA) with random hexamers and RNase inhibitor. RT-qPCR was performed with the ABI PRISM 7900HT Sequence Detection System (Applied Biosystems) using PCR Power SYBR Green PCR Master Mix (Applied Biosystems) and the following primers for human lamin A: primers 1 forward 5′-ATGATCGCTTGGCGGTCTAC-3′, reverse 5′-GCCCTGCGTTCTCCGTTT-3′; primers 2 forward 5′-AATGATCGCTTGGCGGTCTA-3′, reverse 5′-GCCCTGCGTTCTCCGTTT-3′ and primers 3 forward 5′-TGCGCAACAAGTCCAATGAG-3′, reverse 5′-TCCATTCTGGCGCTTGATC-3′ and for human ACTB: forward5′-CACCCAGCACAATGAAGAT-3′, reverse5′-CAAATAAAGCCATGCCAAT-3′.

Gene expression was quantified relative to the housekeeping gene ACTB (β-actin) as an internal control, and results were analyzed by the comparative Ct method using Biogazelle qBasePLUS. Results from technical replicates were represented as the fold increase relative to the mean result.

### Flow Cytometry

Asynchronously growing U2OS cells were cotransfected with the following plasmid combinations: CFP-lamin A plus either YFP or YFP-SNX6; GPF-Lamin A plus either CFP-SNX6 or CFP; and HA-Lamin A plus either YFP or YFP-SNX6. Cells were trypsinized, washed twice in PBS, and collected by centrifugation for 10 min at 300g_va_. After fixing in 4% PFA/2% sucrose for 20 min, cells were washed with 1% BSA/PBS. HA-Lamin A-transfected cells were incubated with anti-HA mouse monoclonal antibody as described for confocal microcopy. To assess the role of RAN and ER tubule-forming proteins in SNX6-dependent lamin A incorporation into the nucleus, nuclei were isolated from U20S cells by treatment with Vindelov solution (3.4 mM Tris, 0.1% NP-40, 0.01 M NaCl) [47]. Cells were examined with a FACSCanto II or a LSRFortessa flow cytometer (BD Biosciences) and data were analyzed with BD FACSDIVA (BD Biosciences) or FlowJo 7.6 (FlowJo Inc).

### Immunoprecipitation

Cell lysates from HA-lamin A-transfected U2OS cells, MEFs and non-transfected U2OS cells were prepared by sonication in ice-cold lysis buffer (20 mM Tris-HCl at pH 7.0, 1% NP-40, 150 mM NaCl, 10% glycerol, 10 mM EDTA, 20 mM NaF, 5 mM sodium pyrophosphate, 1 mM Na_3_VO_4_, 1 mM PMSF). Lysates were precleared with protein A agarose beads (Sigma) and incubated overnight with 3 µg of anti-GFP or anti-lamin A/C antibodies, or with anti-UCP2 and anti-SP1 as negative controls. Antibody-protein complexes were isolated using 40 µL of a 25% w/v suspension of protein A agarose beads. Beads were washed twice with 1% NP-40/PBS and twice with TNE (10 mM Tris-HCl at pH 7.5, 500 mM NaCl, 1 mM EDTA). Proteins were eluted from beads by boiling in Laemmli buffer and analyzed by Western blot.

### Immunoblotting

Whole cell extracts prepared as above were centrifuged for 10 min at 2500g_va_ to remove cell debris and nuclei. Whole lysates were separated by SDS-PAGE, transferred to PVDF membranes (Immobilon-P; Millipore) and probed with the indicated primary antibodies in Tris-buffered saline–Tween 20. Bound antibodies were reacted with horseradish peroxidase secondary antibodies and membranes were developed by enhanced chemiluminescence with Super-Signal West Pico or Femto chemiluminescent substrate (Pierce Chemical).

### Subcellular fractionation

ER fractions were prepared as described previously with minor modifications [48]. Briefly, four 100-mm plates of U20S cells were washed twice with 10 ml PBS containing 136.9 mM NaCl, 2.7 mM KCl, 8.0 mM Na_2_HPO_4_, and 1.5 mM KH_2_PO_4_. After a 5-min incubation in PBS supplemented with 5 mM Na_2_EDTA, cells were scraped off the plate with a rubber policeman, pelleted for 3 min at room temperature at 1000*g*_av_, and resuspended in 2 ml ice-cold hypotonic lysis buffer containing 50 mM sucrose, 10 mM Hepes, pH 7.4, Complete and Phospho-Stop (Roche). Cells were homogenized by 25 strokes of a Dounce homogenizer with a tight pestle. After addition of 264 ml 65% sucrose [(w/w) in 10 mM Hepes, pH 7.4], 4 ml 0.5 M MgCl_2_, and 13.2 ml 2.5 mg/ml aprotinin, the homogenate was subjected to two 10-min spins at 1,000*g*_av_, 47°C to pellet nuclei, mitochondria, and unlysed cells. Crude membranes were pelleted from the resulting supernatant by centrifugation at 100,000*g*_av_ for 30 min at 47°C, washed quickly in 2 ml hypotonic lysis buffer, and resuspended in the same buffer. The membranes were flash frozen in aliquots to avoid freeze-thawing and stored at −80°C for future use. Protein concentration was determined as described previously [48].

The crude membrane pellet from four 100-mm plates of U20S cells was resuspended in 0.7 ml of 10 mM Hepes, pH 7.4, with 15 strokes of a Thomas 0448 Teflon pestle homogenizer, combined with 2.3 ml of 65% (w/w) sucrose in 10 mM Hepes, pH 7.4, and placed at the bottom of an SW41 centrifuge tube. The sample was then sequentially overlaid with 1 ml each of the following sucrose solutions: 45, 40, 35, 30, 25, 20, 15, and 10% (w/w in 10 mM Hepes, pH 7.4). After centrifugation at 47°C for 18 h at 84,000*g*_av_, 0.75-ml fractions were collected from the bottom of the tube, diluted in 6 ml ice-cold PBS containing 16.5 mg/ml aprotinin, and spun for 1 h at 100,000*g*_av_ at 47°C to pellet the membranes. For immunoblot analysis, the membrane pellets were solubilized directly in 100 ml reducing sample buffer.

### In situ nuclear matrix isolation and indirect immuno fluorescence analysis

In situ isolation of nuclear matrix was performed as described previously with minor modifications [49]. Briefly, cells grown on coverslips were washed in PBS and extracted twice in cytoskeleton buffer (CSK: 100 mM NaCl, 300 mM sucrose, 10 mM PIPES (pH 6.8), 3 mM MgCl_2_, 0.5% Triton X-100, and 1.2 mM PMSF) for 10 min at 0°C. The resulting soluble fraction was removed. Extraction buffer (250 mM (NH_4_)_2_SO_4_, 300 mM sucrose, 10 mM PIPES (pH 6.8), 3 mM MgCl_2_, 1.2 mM PMSF, and 0.5% Triton X-100) was added to the Triton X-100 insoluble structures for 10 min at 0°C and the cytoskeleton fraction was removed. DNase digestion was performed twice in digestion buffer (100 µg/ml DNase I and 50 mM NaCl in CSK buffer), followed by extraction in digestion buffer containing 0.25 M (NH_4_)_2_SO_4_. In-situ–extracted and control cells were fixed in 4% formaldehyde in PBS and permeabilized with 0.5% Triton X-100. All samples were blocked for 5 min with 10 mM glycine (pH 8.5) and for 1 h with 5% dry milk in 10% FBS, 0.5% BSA, 0.1% Triton X-100 in PBS, followed by an overnight incubation at 4°C with anti-c-Fos (1∶100), anti-ERK2 (1∶100), or anti-Sp1 (1∶100) antibodies. Samples were then incubated with species-appropriate FITC-conjugated secondary antibodies. After washes and incubation with anti-lamin A/C (1∶100; sc-7292) for 1 h at room temperature, specimens were washed and incubated with an anti–mouse secondary antibody conjugated to Alexa 633 (1∶300) for indirect immunofluorescence analysis.

### Statistical analysis

Results are represented as mean±SE. In experiments with two groups, differences were evaluated using a two-tailed, unpaired Student's *t* test. One-way ANOVA and Bonferroni's post hoc test were used for experiments involving more than two groups.

## Results

### Lamin A/C interacts with SNX6 *in vitro* and *in vivo*

**Figure 1:**
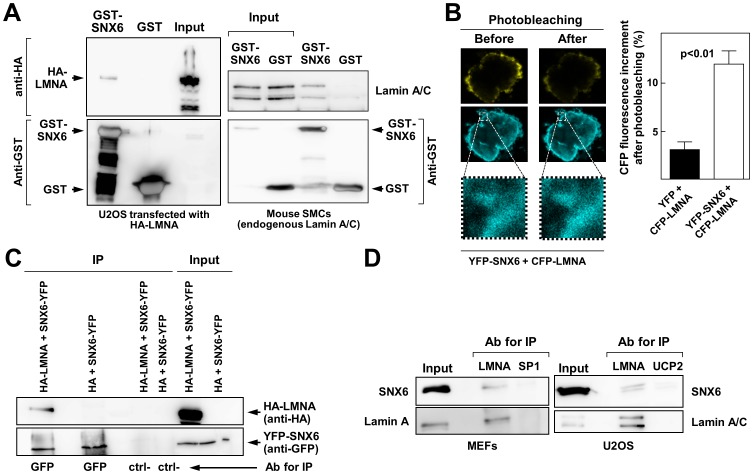
SNX6 interacts with lamin A/C *in vitro* and *in vivo*. (**A**) Cell extracts from U2OS cells overexpressing HA-lamin A (left) or mouse smooth muscle cells (SMCs) expressing endogenous lamin A (right) were subjected to pull-down with GST-SNX6 or GST alone. Pelleted material was probed by immunoblot with the indicated antibodies. Input lane corresponds to an aliquot of the total protein mixture before each pull down experiment. (**B**) *In vivo* interaction between SNX6 and lamin A was quantified by fluorescence resonance energy transfer (FRET) using the acceptor photobleaching method. Data in the graph represent the mean±SE of three independent experiments. The images show a representative example of cells cotransfected with YFP-SNX6 and CFP-LMNA before and after YFP photobleaching. (**C**) U2OS cells were transiently transfected with YFP-SNX6 together with either HA-lamin A or HA alone as indicated. Cell lysates were immunoprecipitated (IP) with anti-GFP antibodies or control immunoglobulins and immunocomplexes were further analyzed by immunoblotting with anti-HA (top blot) to visualize specific interactions or with anti-GFP (bottom blot) to validate the experimental procedure. Ctrl- indicates the use of unrelated antibodies for immunoprecipitation. (**D**) Interaction between endogenous lamin A and SNX6. Cell extracts from mouse embryonic fibroblasts (MEFs) and U2OS cells (right) were immunoprecipitated with antibodies against lamin A (LMNA) or against unrelated proteins (SP1 and UCP2). Samples were analyzed by Western blot with the indicated antibodies.

To investigate the mechanism by which A-type lamins incorporate into the nuclear lamina, we analyzed the potential interaction of lamin A/C with SNX6, a cargo protein that regulates the trafficking of several proteins to specific subcellular locations [20], [21], [37], [50]–[54]. Bacterially expressed GST-SNX6 fusion protein was purified and used in pull-down experiments with cleared lysates of HA-lamin A-transfected U2OS osteosarcoma cells or mouse smooth muscle cells. Western blot analysis revealed that SNX6 was able to interact with ectopically-expressed HA-lamin A in U2OS cells and with endogenous lamin A/C in smooth muscle cells (Fig. 1A). We also assessed the SNX6-lamin A interaction by acceptor-photobleaching FRET in U2OS cells cotransfected with YFP-SNX6 and CFP-lamin A. CFP fluorescence after photobleaching was significantly higher in these cells than in control cells cotransfected with CFP-lamin A and empty YFP (Fig. 1B). Consistent with these findings, anti-GFP antibodies specifically coimmunoprecipitated ectopically expressed HA-lamin A and YFP-SNX6 (Fig. 1C), and endogenous SNX6 and lamin A were coimmunoprecipitated from lysates of MEFs and human U2OS cells (Fig. 1D) using an anti-lamin A/C antibody for immunoprecipitation endogenous lamin A/C. These findings strongly suggest that ectopic and endogenous SNX6 and lamin A interact directly within the cell.

### SNX6 overexpression alters the subcellular distribution of lamin A and increases its accumulation

**Figure 2:**
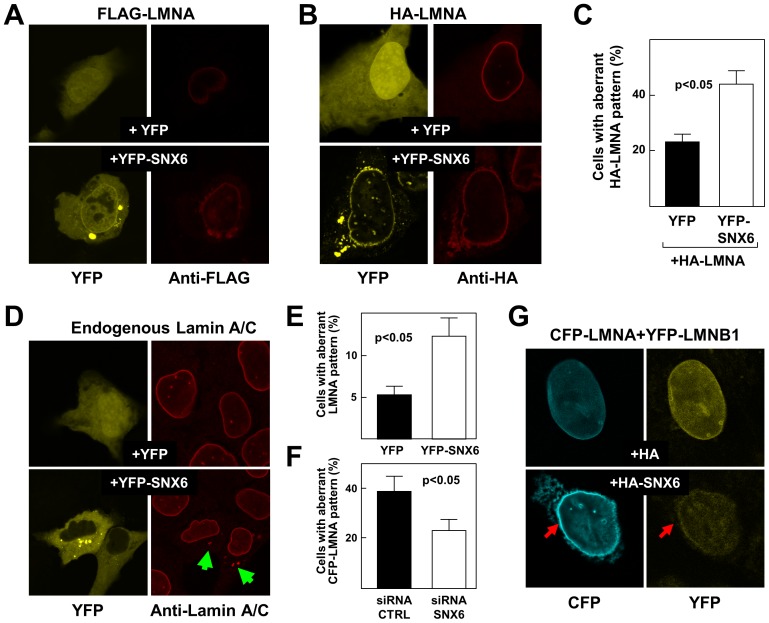
SNX6 overexpression alters lamin A/C subcellular distribution. U2OS cells were transiently transfected as indicated and analyzed by confocal microscopy. (**A**) Cells cotransfected with FLAG-lamin A and YFP localized the mature lamin A protein in the perinucleus (top images, YFP in yellow, FLAG-LMNA in red). Cotransfection of FLAG-LMNA with YFP-SNX6 (bottom images) revealed perinuclear expression of SNX6 together with its high accumulation in external vesicles around the nucleus (left panel, bottom). This co-expression within the cell coincided with the partial re-distribution of FLAG-LMNA into distinctive extra-perinuclear vesicles and the partial loss of the smoothened perinuclear shape. (**B**) Similar results were obtained in cells cotransfected with HA-LMNA and YFP-SNX6. (**C**) Quantification of cells with an aberrant (extranuclear) distribution of HA-lamin A after cotransfection with YFP or YFP-SNX6 (n = 3 independent transfections). (**D**) Cells transfected with YFP alone (top images) or YFP-SNX6 (bottom images) also exhibited an altered distribution of endogenous lamin A/C upon SNX6 overexpression. (**E**) Quantification of cells with an extranuclear expression pattern of endogenous lamin A/C upon transfection with YFP alone or YFP-SNX6 (n = 3 independent transfections). (**F**) Quantification of cells with an extranuclear expression pattern of CFP-lamin A two days after silencing of endogenous SNX6 with specific siRNA (siRNA-SNX6). In controls, cells were transfected with siRNA-CTRL (n = 3 independent transfections). (**G**) Cells were cotransfected with CFP-lamin A, YFP-lamin B1 and either HA alone (top) or HA-SNX6 (bottom). The arrow marks one perinuclear region with significant content of lamin A but no significant changes in the distribution lamin B1. When cotransfected with SNX6, CFP-LMNA, but not YFP-LMNB1, also displays a more diffuse pattern and accumulates in small vesicles.

To further characterize the interaction between SNX6 and lamin A/C, we performed immunofluorescence assays in cells overexpressing tagged proteins. Cells expressing YFP as control showed the typical perinuclear localization of FLAG- and HA-tagged lamin A. However, gross examination of cells overexpressing YFP-SNX6 revealed the typical perinuclear staining of lamin A but also an unexpected cytoplasmic localization (Fig. 2A, B). Quantification of cells with altered lamin A distribution confirmed that overexpression of SNX6 significantly promoted this phenotype (Fig. 2C). Lamin A is expressed as immature pre-lamin A, which is rapidly processed to generate mature lamin A [55]–[57]. To analyze the effect of SNX6 on lamin A localization under more physiological conditions, we overexpressed FLAG-Pre-lamin A in U20S cells. Again, even when the starting protein was FLAG-Pre-lamin A, the final mature FLAG-lamin A exhibited some degree of cytoplasmic localization in the presence of overexpressed SNX6 (S1 Fig.). Interestingly, SNX6 overexpression also significantly increased the accumulation of endogenous lamin A in the cytoplasm (Figs. 2D, 2E). Conversely, the percentage of cells with altered CFP-lamin A distribution was significantly reduced by endogenous SNX6 silencing with small interference RNA (siRNA-SNX6) (Fig. 2F).

The *LMNA* gene encodes lamin A and lamin C (known as A-type lamins). Although lamin C is not farnesylated, it also localizes in the NE, even when lamin A is absent, as observed in a mouse model expressing only lamin C [58]. Transfection experiments showed that HA-SNX6, but not HA alone, also promotes the cytoplasmic localization of ectopically-expressed GFP-lamin C (S1 Fig.). Although mammalian A-type lamins and B-type lamins encoded by the *LMNB1* and *LMNB2* genes share similar structures, processing and localization, these two classes of intermediate filaments form separate networks and have independent behaviors in their incorporation into the nucleus after mitosis [59], [60]. Overexpressed SNX6 had no effect on the subcellular localization of YFP- or CFP-tagged lamin B1 under conditions that promoted lamin A cytoplasmic localization, suggesting that SNX6 specifically regulates A-type lamins (Fig. 2G, S2A-F Fig.). Further supporting the specificity of this interaction, HA-SNX6 failed to alter the subcellular localization of GFP- or YFP-tagged NE-associated (NUP50 and LBR) and soluble (ERK2) proteins (S2G-I Fig.).

**Figure 3:**
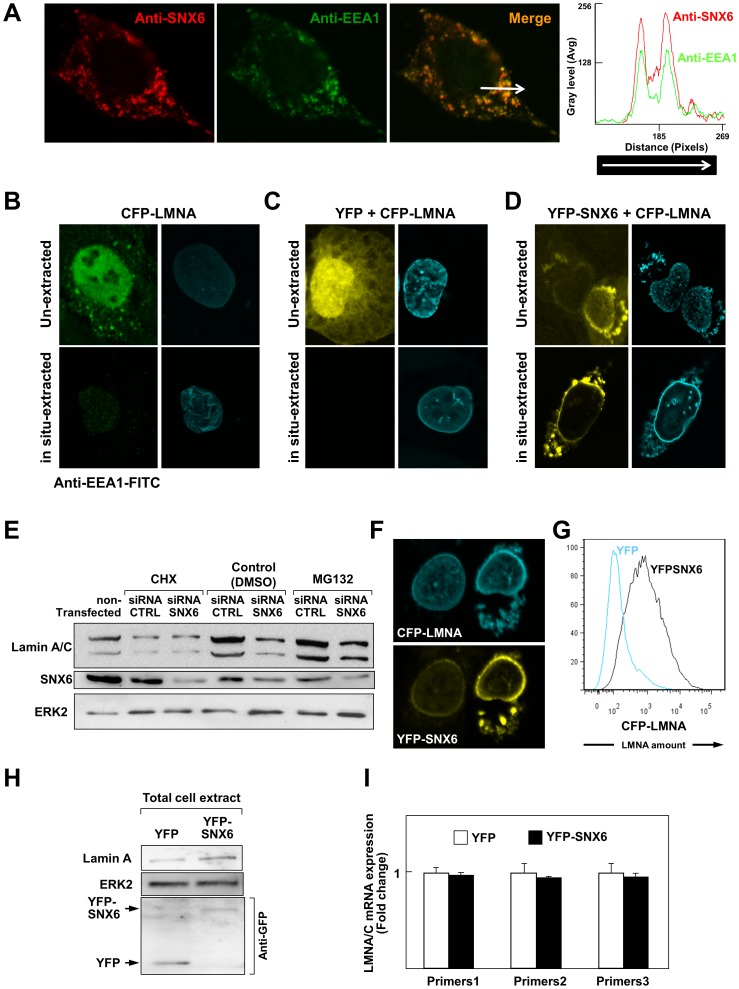
SNX6 and lamin A do not colocalize in early endosomes, and SNX6 overexpression increases the amount of lamin A protein without affecting its proteasomal degradation or mRNA expression. (**A**) Double immunofluorescence confocal microscopy images showing a high degree of colocalization between endogenous SNX6 and EEA1. The graph shows the intensity for each fluorochrome along the arrowed line in the merge image. (**B**) U2OS cells were transfected with CFP-lamin A and two days later were left untreated (top images, un-extracted) or subjected to *in situ* extraction with cytoskeleton buffer (CSK) prior to fixing (lower images, in situ-extracted). Cells were then incubated with FITC-coupled anti-EEA1 antibodies and examined by confocal microscopy. Images reveal the efficient extraction of EEA1 from early endosomes upon in situ extraction with CSK, in contrast to lamin A, which remained in the NE. (**C**) U2OS cells were cotransfected with YFP and CFP-lamin A and treated as in B. Images show the *in situ* extraction of ubiquitously expressed YFP but not of lamin A. (**D**) U2OS cells were cotransfected with YFP-SNX6 and CFP-lamin A and treated as in B. Treatment with CSK did not extract either lamin A or SNX6. (**E**) Western blot analysis of U2OS cells transfected with control siRNA (siRNA-CTRL) or with siRNA targeting SNX6 (siRNA-SNX6). After two days, cultures were treated for 16 h with either cycloheximide (CHX, 10 µg/ml, Sigma), the proteasome inhibitor MG132 (25 µM, Sigma) or vehicle (DMSO). Efficient SNX6 knockdown in cells transfected with siRNA-SNX6 was verified with anti-SNX6 antibody. ERK2 levels were analyzed as a loading control. (**F**) Representative confocal microscopy image showing the correlation between high overexpression of YFP-SNX6 and CFP-lamin A. Pictures were taken of U2OS cells two days after transfection with both plasmids. (**G**) Flow cytometry analysis of cells cotransfected with CFP-lamin A and either YFP or YFP-SNX6, corroborating higher CFP-lamin A expression upon cotransfection of YFP-SNX6. (**H**) Western blot analysis of U2OS cells transfected with YFP or YFP-SNX6 using primary antibodies against lamin A (top), ERK2 (middle) or GFP (bottom). (**I**) RT-qPCR analysis of total RNA isolated from U2OS cells two days post-transfection with either YFP or YFP-SNX6. Relative lamin A/C mRNA levels were determined using three sets of primers (primers1, primers2 and primers3) and calculated relative to values obtained in cells transfected with YFP alone (n = 3 experiments).

SNX6 accelerates the degradation of several proteins by targeting them to the endolysomal proteolytic pathway [37], [61], [62]. We therefore examined whether SNX6 overexpression could affect lamin A transport into early endosomes. As expected, confocal microscopy experiments showed near-complete colocalization of endogenous SNX6 and EEA1 (Fig. 3A). Cell cultures were treated with a cytoskeleton buffer (CSK, see materials and methods) before fixation to gently wash away proteins associated with early endosomes. Although CSK treatment efficiently reduced the amount of endogenous EEA1 (Fig. 3B) and ectopically-expressed YFP (Fig. 3C), it did not affect the content of CFP-lamin A (Figs. 3B, C). Moreover, when coexpressed, YFP-SNX6 and CFP-lamin A displayed a high degree of colocalization and were not washed away by CSK treatment (Fig. 3D). These findings strongly suggest that SNX6 and lamin A do not associate in early endosomes.

Protein degradation in eukaryotic cells is also mediated by the proteasome [63]. To analyze whether SNX6 limits proteasome-dependent degradation of lamin A, we examined the effect of the proteasome inhibitor MG132 on the amount of endogenous lamin A/C upon endogenous SNX6 silencing (Fig. 3E). SNX6-specific siRNA efficiently knocked down SNX6 protein expression in all conditions tested (Fig. 3E), and downregulated the expression of endogenous lamin A/C protein (Fig. 3E, compare siRNA-SNX6 versus siRNA-CTRL in control DMSO-treated cells). MG132 increased the accumulation of lamin A/C in both siRNA-CTRL and siRNA-SNX6 transfected cells. However, MG132 did not prevent siRNA-SNX6-dependent downregulation of lamin A/C (Fig. 3E, compare siRNA-SNX6 versus siRNA-CTRL in MG132-treated cells), suggesting that SNX6 effect on lamin A/C was independent of the proteasome (Fig. 3E). SNX6 overexpression increased the signal intensity of fluorescently-tagged lamin A and C (Figs. 2G, 3F, S1 Fig.), and similar results were obtained in *LMNA*-null MEFs cotransfected with lamin A and SNX6 (S2H Fig.). Therefore, SNX6 might regulate lamin A/C protein expression or turnover. To test this possibility, we cotransfected U2OS cells with CFP-lamin A and either YFP or YFP-SNX6. Flow cytometry analysis of these cells revealed that ectopically-expressed SNX6 increased CFP-lamin A expression (Fig. 3G and S3A Fig.). Moreover, western blot analysis confirmed that YFP-SNX6 overexpression increases the level of both, ectopically expressed CFP-lamin A (S3B Fig., compare the last two lanes in top blot) and endogenous lamin A (Fig. 3H). YFP-SNX6 overexpression did not affect the level of endogenous lamin A/C mRNA (Fig. 3I), suggesting that SNX6-mediated increase of lamin A might be due to increased translation or transport of the protein to the NE. Supporting this notion, treatment with the protein synthesis inhibitor not only reduced the level of lamin A/C but also prevented siRNA-SNX6-dependent downregulation of lamin A/C (Fig. 3E, compare siRNA-SNX6 versus siRNA-CTRL in CHX-treated cells). Together, these results strongly support a role for SNX6 in the expression or stabilization of lamin A protein independent of proteasomal and endolysosomal degradation.

### Cytoplasmic lamin A/C localizes at the surface of the endoplasmic reticulum (ER)

**Figure 4:**
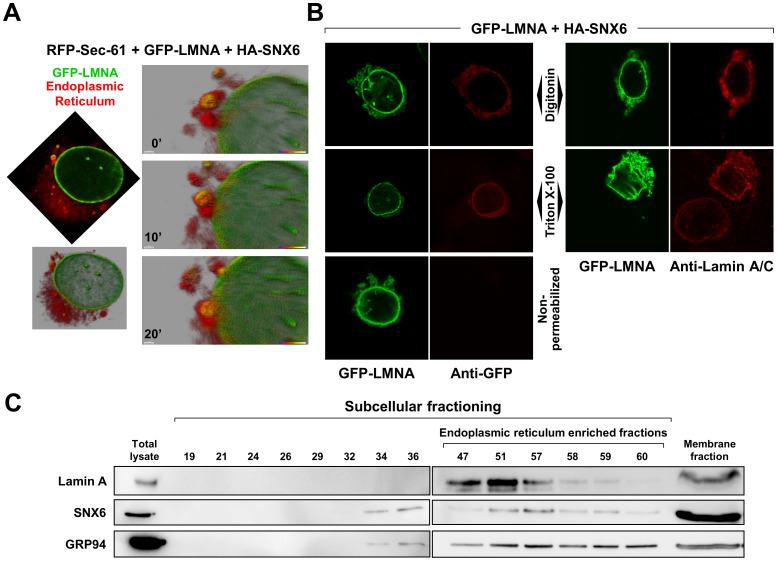
Colocalization of lamin A and SNX6 at the outer surface of the endoplasmic reticulum. (**A**) *In vivo* time-lapse confocal microscopy analysis of U2OS cells cotransfected with GFP-lamin A, HA-SNX6 (to promote extranuclear lamin A accumulation) and RFP-SEC61 (ER label). The top left image shows a representative transfected cell with labeled ER (red) and GFP-lamin A (green) and colocalization of both (yellow). The bottom left image shows an Imaris 3D reconstruction of the same cell. The images on the right show details of 3D reconstructions of the same cell imaged at ten minute intervals. See also S1 Video. (**B**) U2OS cells were transfected with plasmids encoding GFP-Lamin A and HA-SNX6 and processed two days later for immunofluorescence analysis. Cells were non-permeabilized or permeabilized with either Triton X-100 (to permeabilize all membranes) or digitonin (to permeabilize only the plasma membrane). Cells were incubated with anti-GFP antibodies (left) or anti-lamin A/C antibodies (right). Ectopic lamin A was directly visualized by its GFP fluorescence (green) and indirectly from the signals of anti-GFP or anti-lamin A/C antibodies (red). (**C**) Subcellular fractions were prepared from lysates of subconfluent cultures of U2OS cells and the indicated fractions were analyzed by western blot with antibodies against lamin A, SNX6 and the ER marker GRP94.

To identify the subcellular sites at which lamin A/C and SNX6 interact, we cotransfected U2OS cells with YFP-SNX6 and CFP-lamin A and labeled different subcellular compartments with dyes or antibodies targeting specific epitopes. We found no evidence of SNX6 and lamin A colocalization in mitochondria or Golgi apparatus (S4 Fig.). To visualize possible colocalization in the ER, we performed confocal time-lapse analysis of cells cotransfected with GFP-lamin A, HA-SNX6 and RFP-SEC61 (Fig. 4A, and S1 Video). Consistent with the earlier SNX6 overexpression data, lamin A in these cells was detected in the NE and in extranuclear compartments (Fig. 4A, top left image). Three-dimensional reconstruction (Imaris) confirmed the localization of extranuclear lamin A in the ER (yellow staining in Fig. 4A, bottom left). Moreover, sequential images of the same cell over a period of 20 min revealed comigration of extranuclear lamin A and the ER toward the nucleus (Fig. 4A, right). To investigate the localization of lamin A in the ER, we took advantage of the different permeabilization properties of digitonin and Triton X-100. Whereas Triton X-100 permeabilizes all cellular membranes, digitonin selectively permeabilizes the plasma membrane without significantly affecting the gross structure and function of the ER [64]. When cells were cotransfected with GFP-lamin A and HA-SNX6 and permeabilized with either digitonin or Triton X-100 it was possible to detect a signal with anti-GFP antibody, which cannot cross membranes (Fig. 4B, left panels). Similarly, both detergents were equally effective at exposing antigens specific to anti-lamin A/C antibodies (Fig. 4B, right panels). Moreover, western blot of U2OS subcellular fractions confirmed the predominant colocalization of endogenous lamin A and SNX6 in fractions containing the ER marker GRP94 (Fig. 4C). These results thus indicate that SNX6 and lamin A associate at the outer, cytosolic surface of the ER.

### SNX6-dependent nuclear incorporation of lamin A is mediated by Ran

**Figure 5:**
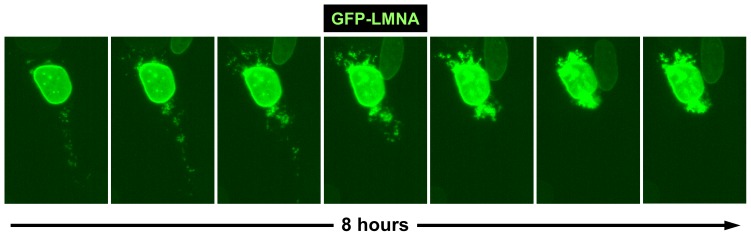
In vivo shuttling of lamin A to the nucleus. Time-lapse analysis of U2OS cells cotransfected with GFP-lamin A and HA-SNX6 to enhance GFP-lamin A extranuclear accumulation. Over a period of 8 hours, the extranuclear GFP-Lamin A progressively incorporated into the nucleus of the transfected cell. See also S2 Video.

**Figure 6:**
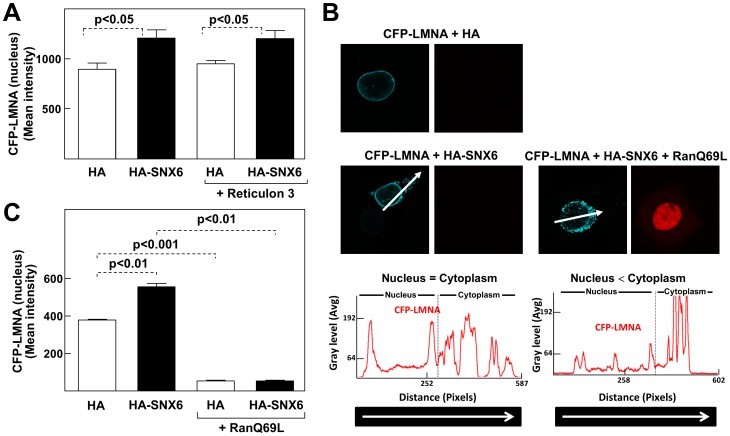
SNX6-dependent lamin A incorporation into the nucleus occurs via a RAN-dependent mechanism and is independent of ER tubule-forming proteins. (**A**) Flow cytometry analysis of nuclei isolated from U2OS cells cotransfected with CFP-lamin A and either HA alone or HA-SNX6. When indicated, cells were also cotransfected with reticulon 3. (**B**) Confocal microscopy analysis of U2OS cells cotransfected as indicated. The intensity of the CFP signal across the cell nucleus and cytoplasm (arrows) is shown for each cell in the graphs below. The arrows across the cells correspond to the sections along which CFP-Lamin A signals were quantified. (**C**) Nuclei from cells transfected as in (B) were isolated and analyzed by flow cytometry.

Our *in vivo* studies with tagged lamin A proteins suggest that SNX6 facilitates the trafficking of lamin A from the ER into the NE. Supporting this idea, time-lapse imaging of U2OS cells cotransfected with HA-SNX6 and GFP-lamin A showed shuttling of GFP-lamin A from the ER to the nucleus (Fig. 5 and S2 Video). To better define the role of SNX6 in this process, we quantified the CFP-lamin A signal intensity in isolated nuclei by flow cytometry. Cotransfection with HA-SNX6 significantly increased the amount of CFP-lamin A in the nucleus (Fig. 6A), suggesting that this process is, at least in part, mediated by SNX6.

Two mechanisms for the incorporation of proteins into the NE are diffusion-retention and targeting with classical NLSs [8], [9]. In diffusion-retention, integral or associated membrane proteins are proposed to reach the ONM by diffusion through ER membranes [10]. Incorporation of proteins to the NE via this route is inhibited by reticulon proteins [42]. However, we found that reticulon 3 overexpression did not inhibit CFP-lamin A nuclear shuttling, suggesting that diffusion-retention is not important for lamin A nuclear import (Fig. 6A). In NLS-mediated NE incorporation, INM proteins are transported to the NE through NPCs, directed by their NLS and a gradient of soluble Ran-GTP/Ran-GDP [12], [13]. To ascertain if SNX6-mediated nuclear import of lamin A requires Ran-GTP/Ran-GDP, we investigated the effect of overexpressing the GTP-bound form of RanQ69L, a dominant-negative mutant which inhibits nuclear import through NPCs [65]. Confocal microscopy analysis across cells overexpressing CFP-lamin A and HA-SNX6 confirmed the accumulation of CFP-lamin A in both the NE and the cytoplasm (Fig. 6B). Importantly, cotransfection with RanQ69L potently inhibited the incorporation of CFP-lamin A into the NE and promoted its accumulation in perinuclear cytoplasmic regions (Fig. 6B). Likewise, flow cytometry analysis of isolated nuclei demonstrated reduced nuclear accumulation of GFP-lamin A upon RanQ69L overexpression, both in control (HA) and in HA-SNX6-overexpressing cells (Fig. 6C). These results reveal that SNX6-dependent nuclear import of lamin A protein occurs through the NPC by a RAN-dependent mechanism.

## Discussion

The mechanisms underlying A-type lamin-dependent regulation of structural and functional processes have been investigated extensively. However, less is known about the mechanisms and cofactors that regulate lamin A/C nuclear import and its integration into the NE to maintain nuclear lamina homeostasis. Our results provide the first demonstration that SNX6 and lamin A proteins interact in mammalian cells, and that SNX6 contributes to regulate lamin A protein content. We have also shown that SNX6 links lamin A to the outer surface of the ER during trafficking to the nucleus, and lamin A nuclear import induced by SNX6 occurs across the NPC through a Ran-dependent mechanism.

Previous studies have established that SNX6 contributes to retrograde endosome-to-Golgi protein transport by linking the dynein-dynactin motor to retromer-associated membranous cargo [20], [50]. In addition, it is well known that SNX6 interacts with several proteins to regulate their final destination in the cell [20], [37], [50], [54], [61], [62]. For example, SNX6 interacts with and inhibits signaling by the TGF-β family of Ser-Thr kinase receptors [61], promotes degradation of the epidermal growth factor receptor through interactions with the G-protein coupled receptor kinase-2 interacting protein 1 [62], and interacts with and targets the tumor suppressor p27^Kip1^ to endolysosomal degradation [37]. Our findings indicate that SNX6 increases lamin A/C protein levels and its accumulation in the NE. SNX6 might increase A-type lamin transport to the nucleus and/or protein translation, since the effects of SNX6 occurred without changes in *LMNA* mRNA levels or lamin A/C degradation and were abrogated upon protein synthesis inhibition with cycloheximide. In our experiments, SNX6 overexpression allowed visualization of accumulated lamin A/C in transit to the nucleus. This accumulation could be an artefact resulting from the formation of aggregates of misfolded lamin A/C. However, this possibility seems unlikely since polypeptides that do not pass ER quality control and cannot be rescued are subsequently targeted to the proteosomal or endolysosomal compartments for degradation [66]. In our experiments, SNX6 overexpression directed lamin A/C to the nucleus and facilitated its accumulation but did not direct it to the endolysosomal or proteosomal compartments, indicating that the extranuclear lamin A/C accumulation reflects normal cellular processing.

Our studies indicate that the effects of SNX6 on lamin A/C subcellular distribution are specific, since SNX6 did not affect the localization of other nuclear factors, including soluble proteins (ERK2) and NE-associated proteins (NUP50, LBR and lamin B1). The lack of effect of SNX6 on lamin B1 localization is in agreement with previous studies demonstrating that A- and B-type lamins form separate networks in the nuclear lamina [59], [60] and coincides with the independent behaviors of A- and B-type lamins during their nuclear incorporation after mitosis. During cell division, the NE is completely disassembled and A-type lamins are released to the nucleoplasm [67] followed by B-type lamins [68] at the transition from prophase to prometaphase. During NE reassembly, A-type lamins incorporate into the NE independently of B-type lamins but after the assembly of other major NE components, including the NPCs [69], [70]. A-type lamins that remain in the cytoplasm are transported into the nucleus after enclosure of the chromatin and formation of an intact NE. Newly-synthesized lamins are transported through NPCs and continue to be incorporated into the NE of the interphase nucleus [71]. Lamin A is synthesized as prelamin A, which undergoes a series of posttranslational modifications in its C-terminal end (farnesylation, carboxymethylation and proteolytic cleavage) to generate mature lamin A within two hours of synthesis [55]–[57]. Although some of our experiments involve overexpressed lamin A, the effects of SNX6 on the localization and amount of lamin A were confirmed in experiments with overexpressed prelamin A.

Proteins are synthesized in the cytoplasm and some enter the ER for maturation [66]. Our selective permeabilization experiments indicate, however, that A-type lamins do not enter the ER, but remain associated with the outer ER surface. A previous study reported association of SNX14 with the ER [72], and SNX6 could therefore be the link of the lamin A/C to the ER. We also discarded a possible interaction between lamin A/C and SNX6 in mitochondria or Golgi apparatus. Consistent with our findings indicating that SNX6 and lamin A travel together to the ER before lamin A nuclear import, enzymes known to be required for prelamin A maturation have been detected both in the INM [73] and in the ER [74].

Among the four mechanisms that have been proposed to regulate the transport of proteins to the INM [8], [9] (see Introduction), the diffusion-retention model suggests that integral membrane proteins synthesized in the ER reach the ONM by diffusion through the ER membranes [10], with subsequent transfer from the ONM to the INM occurring by passive lateral diffusion at the sites of NPC insertion [11]. On the other hand, the NLS-targeting model proposes that INM proteins are transported to their final destination after recognition of an NLS by importins and karyopherins, which in turn interact with the NPCs and then transport INM proteins to the nuclear interior along gradients of soluble Ran-GTP/Ran-GDP created by Ran-GTPases [12], [13]. Our analysis shows that overexpression of reticulon 3, which inhibits diffusional transport of proteins from the ER to the NE [42], does not affect SNX6-mediated increased incorporation of lamin A/C into the NE, suggesting that lateral diffusion does not mediate lamin A/C nuclear transport. In contrast, overexpression of a dominant-negative form of RAN-GTP, which blocks NLS-dependent nuclear import of proteins across NPCs, inhibited nuclear accumulation of lamin A/C and increased their localization in the cytoplasm in association with the ER. These results are in accordance with previous studies demonstrating that importin α/β recognizes the NLS of lamin A/C to facilitate internalization across the NPCs before its association with the INM and incorporation into the nuclear lamina [75], [76]. Our findings identify SNX6 as a key regulator of lamin A synthesis and transport to the nucleus, revealing a novel mechanism for specific cytoplasmic transport of lamin A prior to its nuclear import via RAN-GTP. Future studies are warranted to examine the regulation of SNX6-lamin A/C interaction and its functional consequences in different pathophysiological scenarios.

## Supporting Information

S1 Fig **SNX6 overexpression affects lamin A/C distribution.** U2OS cells were cotransfected with the indicated vectors and analyzed by confocal microscopy. (**A**) Cells were transfected with FLAG-Pre-lamin A together with HA alone (top) or HA-SNX6 (bottom). (**B**) Cells were transfected with FLAG-Pre-lamin A together with either YFP (top) or YFP-SNX6 (bottom). Cotransfection with HA-SNX6 or YFP-SNX6 caused accumulation of lamin A in cytoplasmic regions and increased the intensity of the lamin A signal. (**C**) Cells were transfected with GFP-lamin C together with HA alone (top) or HA-SNX6 (bottom). HA-SNX6 caused extraperinuclear relocalization and increased intensity of the signal for lamin C.
(TIF)

S2 Fig **Overexpression of SNX6 specifically alters the cellular distribution of lamin A.** Cells were transfected as indicated and examined by confocal microscopy. (**A**) YFP-SNX6 does not alter CFP localization. (**B**) Cotransfection of YFP-LMNB1 and CFP-LMNB1 to show YFP-LMNB1 and CFP-LMNB1 localization pattern. (**C**) CFP-lamin A localization pattern is altered by overexpression of YFP-SNX6 but not YFP. (**D**) CFP-lamin B1 localization is not altered by coexpression of YFP-SNX6 or YFP. (**E**) YFP-lamin B1 localization is not altered by coexpression of CFP-SNX6 or CFP. (**F**) Percentage of cells with an aberrant (extranuclear) distribution of GFP-lamin A or GFP-Lamin B1 upon coexpression of CFP alone or CFP-SNX6. (**G**) HA-SNX6 overexpression in U2OS cells alters the subcellular localization of CFP-lamin A without affecting the distribution of the NE-associated protein NUP50 (GFP-NUP50). (**H**) In Lmna-KO MEFs, overexpression of HA-SNX6, but not of HA, alters the subcellular localization of CFP-LMNA without affecting NE-associated protein the distribution of the NE-associated protein Lamin B Receptor (YFP-LBRTM1). (**I**) Confocal microscopy analysis of U2OS cells transfected with HA-SNX6 (left), GFP-ERK2+HA (middle) or GFP-ERK2+HA-SNX6 (right), showing lack of effect of HA-SNX6 on the distribution of GFP-ERK2. HA-SNX6 was revealed with anti-HA antibody and a fluorescently labeled secondary antibody.
(TIF)

S3 Fig **SNX6 overexpression increases lamin A protein levels.** (A) Flow cytometry analysis of U2OS cells transfected with GFP-lamin A plus either CFP or CFP-SNX6 (left) or with HA-lamin A plus either YFP or YFP-SNX6 (right). Expression of the HA epitope was detected with APC-linked anti-HA secondary antibodies. In both experiments, SNX6 overexpression increased the signal for lamin A, shown by the rightward shift in cells expressing CFP-SNX6 or YFP-SNX6. (B) Western blot analysis of whole-cell lysates from U2OS cells transfected with the vectors indicated. Fluorescent proteins were detected with anti-GFP antibody and identified based on their different electrophoretic motilities. Ectopic overexpression of SNX6 resulted in overexpression of lamin A (compare the two last lanes on the right).
(TIF)

S4 Fig **SNX6 and lamin A do not colocalize in mitochondria or the Golgi apparatus.** Confocal microscopy analysis of U2OS cells cotransfected and treated as follows: *Top*: Cotransfection with CFP-lamin A, HA-SNX6 and YFP-Frataxin MLS (mitochondrial localization signal of frataxin, to visualize mitochondria). *Center*: Cotransfection with HA-lamin A, CFP-SNX6 and eNOS-GFP (to visualize the Golgi apparatus). *Bottom*: Cotransfection with CFP-lamin A and HA-SNX6 plus treatment with Bodipy TR ceramide to identify the Golgi apparatus. Graphs show the intensities for each fluorochrome along the path marked by the arrow in the merge images.
(TIF)

S1 Video **Localization of lamin A at the endoplasmic reticulum upon the overexpression of SNX6.** (**A**) Imaris 3D reconstruction of i*n vivo* time-lapse confocal microscopy analysis of U2OS cells cotransfected with GFP-lamin A, HA-SNX6 (to promote extranuclear lamin A accumulation) and RFP-SEC61 (ER label). ER (red) and GFP-lamin A (green) and colocalization of both (yellow). U2OS cells cotransfected with RFP-Sec-61, GFP-Lamin A and HA-SNX6 were examined under a TCS SP5 confocal laser scanning unit attached to an inverted epifluorescence microscope (DMI6000) fitted with an HCX PL APO 63/NA 1.40-0.60 oil objective. Cells were maintained in DMEM (containing 10%FBS and 20 mM Hepes) in 35 mm dishes (MatTek) at 37°C in a 5% CO_2_ atmosphere.
(MPG)

S2 Video **In vivo shuttling of lamin A to the nucleus.** Time-lapse analysis of U2OS cells cotransfected with GFP-lamin A and HA-SNX6 to enhance GFP-lamin A extranuclear accumulation. Over a period of 8 hours, the extranuclear GFP-Lamin A progressively incorporated into the nucleus of the transfected cell. U2OS cells cotransfected with GFP-Lamin A and HA-SNX6 were examined under a Nikon ECLIPSE Ti time-lapse inverted microscope fitted with an 40× air objective (NA 0.6) using filters for GFP Cells were maintained in DMEM (containing 10%FBS and 20 mM Hepes) in 35 mm dishes (MatTek) at 37°C in a 5% CO_2_ atmosphere.
(AVI)
