# Supplementary figures and images for: Sorting Nexin 6 Enhances Lamin A Synthesis and Incorporation into the Nuclear Envelope
Source: PLoS One. 2014 Dec 23;9(12):e115571. doi: 10.1371/journal.pone.0115571 (PMC4275242; doi:10.1371/journal.pone.0115571)

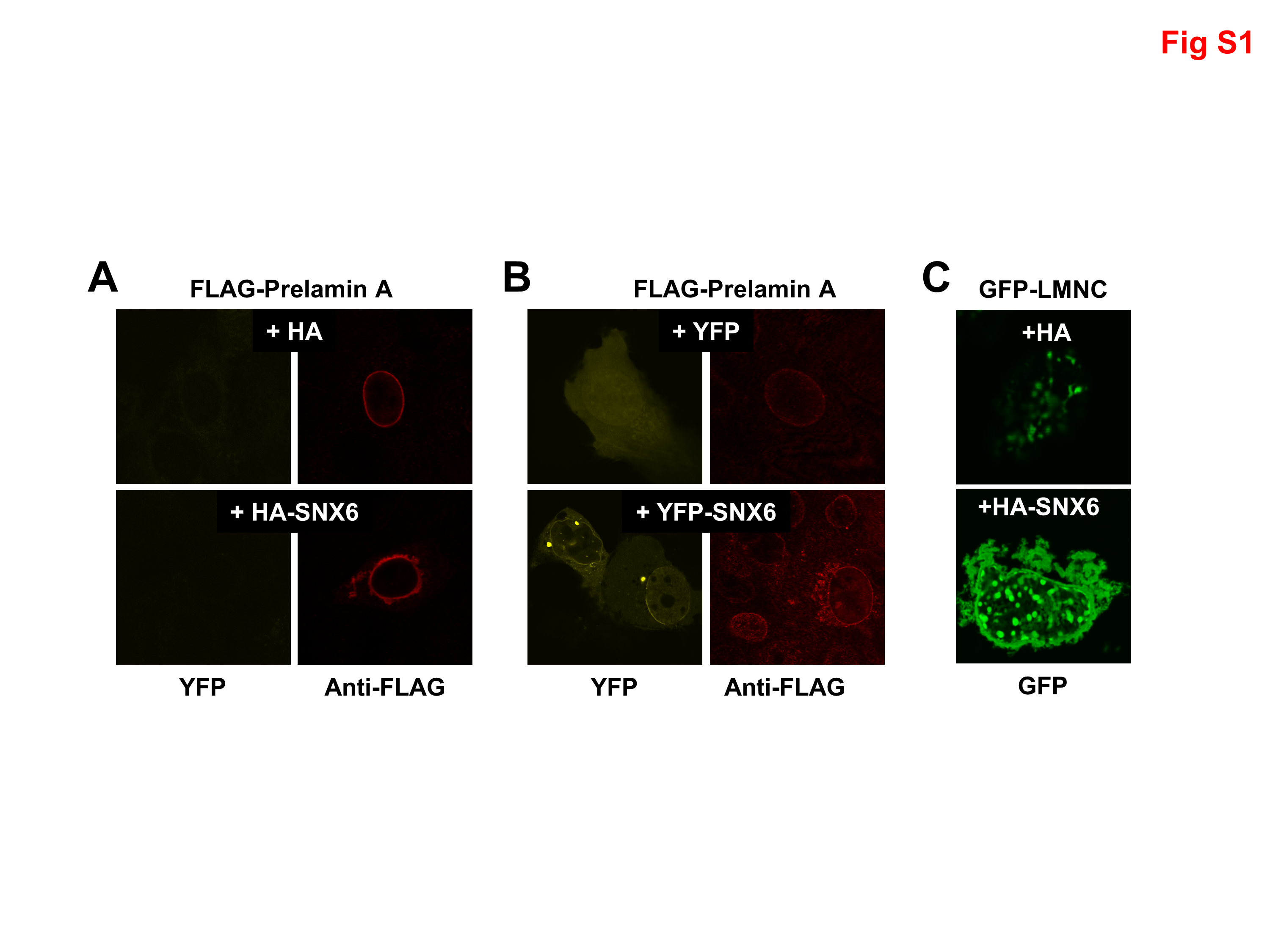

Supplement: S1 Fig — SNX6 overexpression affects lamin A/C distribution. U2OS cells were cotransfected with the indicated vectors and analyzed by confocal microscopy. (A) Cells were transfected with FLAG-Pre-lamin A together with HA alone (top) or HA-SNX6 (bottom). (B) Cells were transfected with FLAG-Pre-lamin A together with either YFP (top) or YFP-SNX6 (bottom). Cotransfection with HA-SNX6 or YFP-SNX6 caused accumulation of lamin A in cytoplasmic regions and increased the intensity of the lamin A signal. (C) Cells were transfected with GFP-lamin C together with HA alone (top) or HA-SNX6 (bottom). HA-SNX6 caused extraperinuclear relocalization and increased intensity of the signal for lamin C. (TIF) [file pone.0115571.s001.tif]

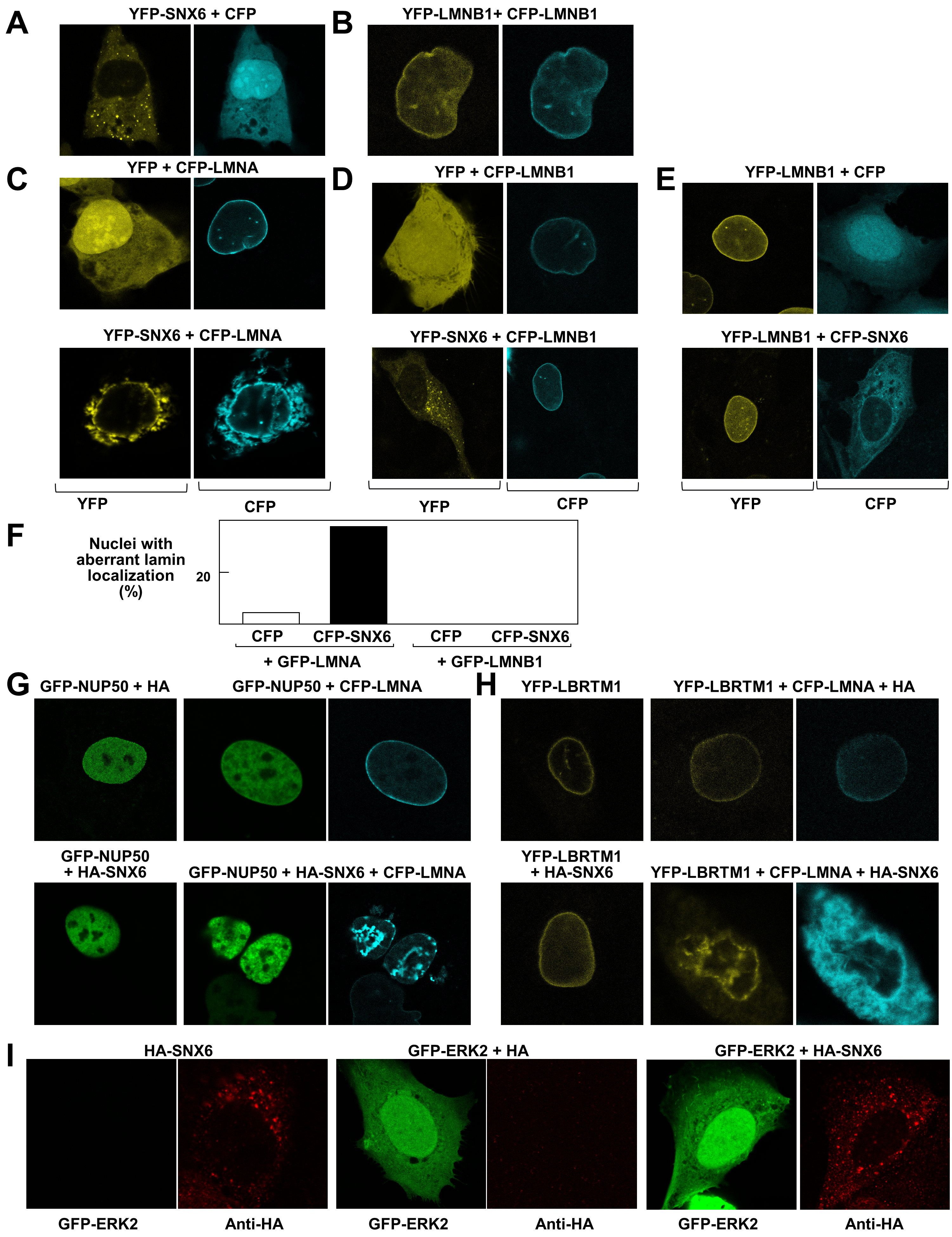

Supplement: S2 Fig — Overexpression of SNX6 specifically alters the cellular distribution of lamin A. Cells were transfected as indicated and examined by confocal microscopy. (A) YFP-SNX6 does not alter CFP localization. (B) Cotransfection of YFP-LMNB1 and CFP-LMNB1 to show YFP-LMNB1 and CFP-LMNB1 localization pattern. (C) CFP-lamin A localization pattern is altered by overexpression of YFP-SNX6 but not YFP. (D) CFP-lamin B1 localization is not altered by coexpression of YFP-SNX6 or YFP. (E) YFP-lamin B1 localization is not altered by coexpression of CFP-SNX6 or CFP. (F) Percentage of cells with an aberrant (extranuclear) distribution of GFP-lamin A or GFP-Lamin B1 upon coexpression of CFP alone or CFP-SNX6. (G) HA-SNX6 overexpression in U2OS cells alters the subcellular localization of CFP-lamin A without affecting the distribution of the NE-associated protein NUP50 (GFP-NUP50). (H) In Lmna-KO MEFs, overexpression of HA-SNX6, but not of HA, alters the subcellular localization of CFP-LMNA without affecting NE-associated protein the distribution of the NE-associated protein Lamin B Receptor (YFP-LBRTM1). (I) Confocal microscopy analysis of U2OS cells transfected with HA-SNX6 (left), GFP-ERK2+HA (middle) or GFP-ERK2+HA-SNX6 (right), showing lack of effect of HA-SNX6 on the distribution of GFP-ERK2. HA-SNX6 was revealed with anti-HA antibody and a fluorescently labeled secondary antibody. (TIF) [file pone.0115571.s002.tif]

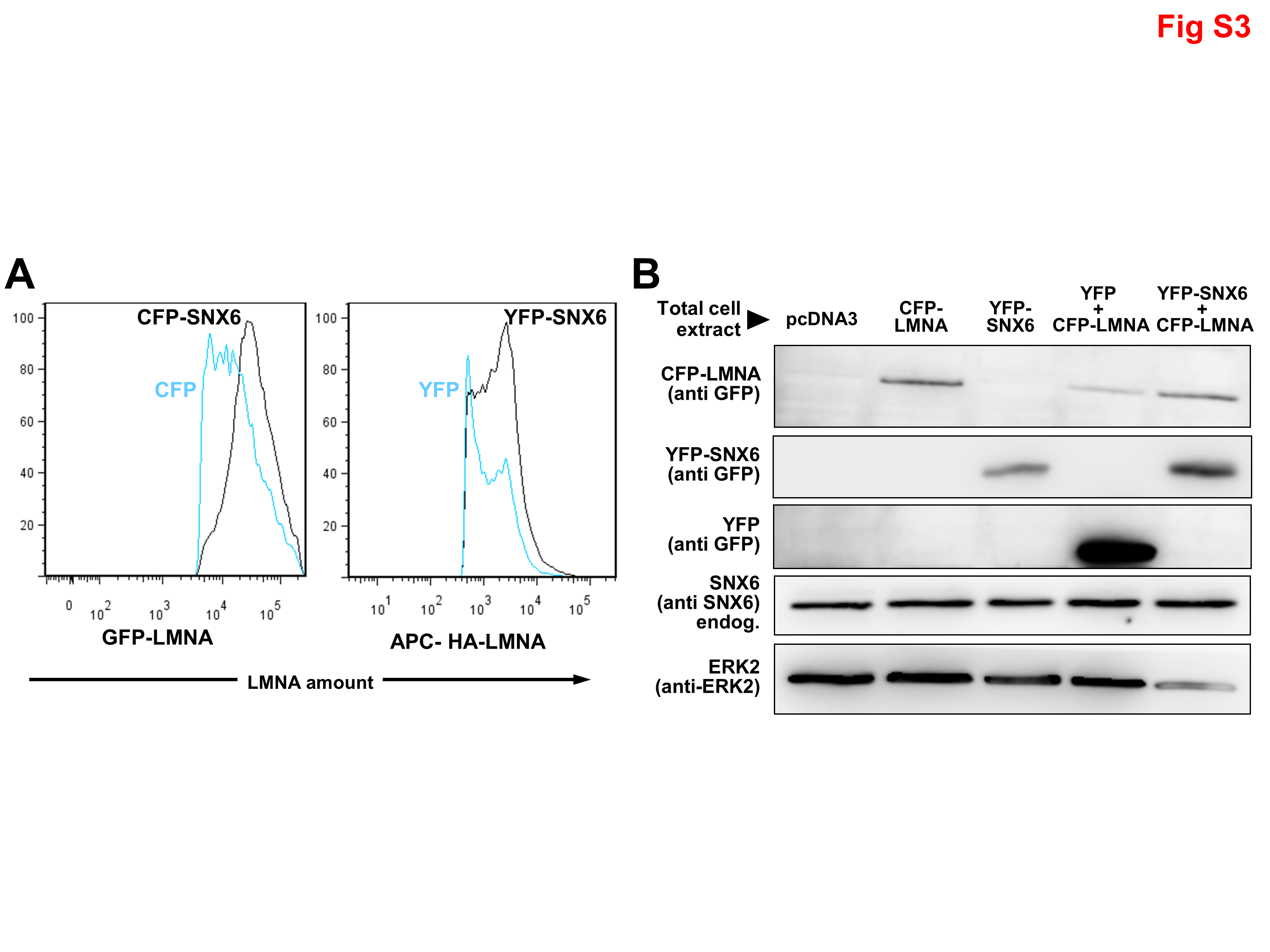

Supplement: S3 Fig — SNX6 overexpression increases lamin A protein levels. (A) Flow cytometry analysis of U2OS cells transfected with GFP-lamin A plus either CFP or CFP-SNX6 (left) or with HA-lamin A plus either YFP or YFP-SNX6 (right). Expression of the HA epitope was detected with APC-linked anti-HA secondary antibodies. In both experiments, SNX6 overexpression increased the signal for lamin A, shown by the rightward shift in cells expressing CFP-SNX6 or YFP-SNX6. (B) Western blot analysis of whole-cell lysates from U2OS cells transfected with the vectors indicated. Fluorescent proteins were detected with anti-GFP antibody and identified based on their different electrophoretic motilities. Ectopic overexpression of SNX6 resulted in overexpression of lamin A (compare the two last lanes on the right). (TIF) [file pone.0115571.s003.tif]
